# Novel Homozygous Missense Mutation in the *ARG1* Gene in a Large Sudanese Family

**DOI:** 10.3389/fneur.2020.569996

**Published:** 2020-10-29

**Authors:** Liena E. O. Elsayed, Inaam N. Mohammed, Ahlam A. A. Hamed, Maha A. Elseed, Mustafa A. M. Salih, Ashraf Yahia, Rayan Abubaker, Mahmoud Koko, Amal S. I. Abd Allah, Mustafa I. Elbashir, Muntaser E. Ibrahim, Alexis Brice, Ammar E. Ahmed, Giovanni Stevanin

**Affiliations:** ^1^Faculty of Medicine, University of Khartoum, Khartoum, Sudan; ^2^College of Medicine, Princess Nourah Bint Abdulrahman University, Riyadh, Saudi Arabia; ^3^Institut du Cerveau, INSERM, CNRS, Sorbonne Université, Paris, France; ^4^Division of Pediatric Neurology, Department of Pediatrics, College of Medicine, King Saud University, Riyadh, Saudi Arabia; ^5^Department of Biochemistry, Faculty of Medicine, National University, Khartoum, Sudan; ^6^Ecole Pratique des Hautes Etudes, EPHE, PSL Research University, Paris, France; ^7^Department of Molecular Biology, Institute of Endemic Diseases, University of Khartoum, Khartoum, Sudan; ^8^Department of Neurology and Epileptology, Hertie Institute for Clinical Brain Research, Tuebingen, Germany; ^9^APHP, Pitié-Salpêtrière Hospital, Department of genetics, Paris, France

**Keywords:** hyperargininemia, ARG1 gene, whole exome sequencing, Sudan, spastic quadriplegia

## Abstract

**Background:** Arginases catalyze the last step in the urea cycle. Hyperargininemia, a rare autosomal-recessive disorder of the urea cycle, presents after the first year of age with regression of milestones and evolves gradually into progressive spastic quadriplegia and cognitive dysfunction. Genetic studies reported various mutations in the *ARG1* gene that resulted in hyperargininemia due to a complete or partial loss of arginase activity.

**Case Presentation:** Five patients from an extended highly consanguineous Sudanese family presented with regression of the acquired milestones, spastic quadriplegia, and mental retardation. The disease onset ranged from 1 to 3 years of age. Two patients had epileptic seizures and one patient had stereotypic clapping. Genetic testing using whole-exome sequencing, done for the patients and a healthy parent, confirmed the presence of a homozygous novel missense variant in the *ARG1* gene [GRCh37 (NM_001244438.1): exon 4: g.131902487T>A, c.458T>A, p.(Val153Glu)]. The variant was predicted pathogenic by five algorithms and affected a highly conserved amino acid located in the protein domain ureohydrolase, arginase subgroup. Sanger sequencing of 13 sampled family members revealed complete co-segregation between the variant and the disease distribution in the family in line with an autosomal-recessive mode of inheritance. Biochemical analysis confirmed hyperargininemia in five patients.

**Conclusion:** This study reports the first Sudanese family with *ARG1* mutation. The reported variant is a loss-of-function missense mutation. Its pathogenicity is strongly supported by the clinical phenotype, the computational functional impact prediction, the complete co-segregation with the disease, and the biochemical assessment.

## Background

Arginases (EC 3.5.3.1) are proteins involved in the last reaction of the urea cycle in which they catalyze the hydrolysis reaction of l-arginine to form urea and ornithine. There are two isoforms of the arginase enzyme (arginases 1 and 2) encoded by the *ARG1* [^*^608313] and *ARG2* [^*^107830] genes, respectively. Arginase one predominates in the liver, constituting 98% of the hepatic arginase activity. Its deficiency results in hyperargininemia/arginase deficiency [#207800], which is an autosomal-recessive (AR) inborn error of metabolism. Arginase deficiency is the least common of all urea cycle disorders. The incidence of hyperargininemia has been estimated to vary between 1:350,000 and 1:1,000,000 (1). There is scarcely any data available about the incidence/prevalence in Sudan and other Sub-Saharan African countries.

Most commonly, the condition manifests clinically between 1 and 3 years of age, although adult-onset arginase deficiency has been occasionally reported (2). Classically, patients suffer from developmental delay and regression of the acquired milestones. The condition may then evolve gradually into spastic quadriparesis and intellectual disability if left untreated (3). The condition can be almost non-progressive, which can sometimes lead to confusion with cerebral palsy (4). Biochemically, there is hyperargininemia with periodic hyperammonemia, although, in contrast to other urea cycle disorders, neonatal hyperammonemia and its clinical features are relatively uncommon in arginase deficiency. Affected children are frequently asymptomatic at birth and in their early childhood.

The *ARG1* gene is located on chromosome six mapping to the cytogenetic location 6q23.2. The gene harbors eight exons. Hyperargininemia is associated with pathogenic variants in both homozygous and compound heterozygous states, with no obvious genotype/phenotype correlation. The first case in whom the genetic basis of argininemia was characterized was a Japanese patient with compound heterozygous mutations (5). A study published in 2018 by Diez-Fernandez et al. carried out sequence analysis compiling 66 published and novel variants of the *ARG1* gene and found that the most encountered category was point mutations (missense, non-sense, and splicing), although other structural alterations were also found. Missense variants were the most common mutation type distributed all over the coding region of the gene (6–8). Genetic studies of hyperargininemia are extremely rare in Sub-Saharan Africa and are non-existent in Sudan. In this study, we are reporting the first case from Sudan with *ARG1* mutation. A novel homozygous missense mutation in the *ARG1* gene was identified in five spastic quadriplegic patients from an extended highly consanguineous Sudanese family.

## Case Presentation

Five affected patients from three branches of family F15 are reported in this study. The patients had a history of regression of milestones following a normal early development till the age at onset, which ranged from 1 to 3 years. The clinical picture was dominated by spastic quadriplegia complicated by cognitive impairment and sphincter disturbances in the majority of patients. Visual and hearing assessments were normal, except in one patient who had conductive deafness in the right ear (patient 138). Despite the remarkable homogeneity of the clinical presentation in all branches, two patients had epileptic seizures (patients 138 and 142) and one patient (patient 138) had stereotypic clapping (Table 1). Extrapyramidal and cerebellar symptoms and signs were not detected in all five patients. No sensory nervous system abnormality was detected, although assessment could not be done properly. Brain images (MRI) were normal in all patients. The disease had a progressive nature, as expected, with variable severity. The most severe course was observed in one patient who died in the first half of the second decade.

**Table 1:** Clinical data of five patients from family F15 with hyperargininemia due to a mutation in the *ARG1* gene.

**Mutated gene**	***ARG1***				
Individual code	138	142	143	148	149
Gender	F	M	M	M	F
Clinical diagnosis	Spastic quadriplegia				
Age at onset of motor symptoms	1 year	3 years	2 years, 9 months	3 years	2 years, 8 months
Age at initial examination	8 years	10 years	6 years	10 years	8 years
Spasticity UL/LL	++/+++	++/++	+++/+++	++/+++	+++/+++
Motor deficit PUL/DUL	+/+	+/+	+/+	+/–	–/–
Motor deficit PLL/DLL	++/++	+/+	++/++	+++/+++	–/–
Tendon reflexes UL/LL patellar	↑/↑	↑/↑	↑/↑	↑/↑	↑/↑
Ankle reflex/ plantar response	↑/↑↑	↑/↑↑	↑/↔	↑/↑↑	↑*↑↑*
Ataxia eye/UL/LL/gait	–/–/–/–	–/–/–/–	–/–/–/–	–/–/–/–	–/–/–/–
Dysarthria spastic/cerebellar	+++/–	+/–	+/–	+++/–	+++/–
Muscle atrophy DUL/DLL	+/+	+/+	–/+	–/+	–/–
Facial atrophy	–	–	–	–	–
Cognitive/psychiatric signs	+/+	+/+	+/+	–/–	+/+
Sensory loss	–	–	–	–	–
Optic atrophy	–	–	–	–	–
Extrapyramidal signs	–	–	–	–	–
Other signs	Severe urinary/anal incontinence Pes cavus/scoliosis Epilepsy Rt. hearing impairment	Severe urinary/anal incontinence Epilepsy	Anal incontinence	Pes cavus/scoliosis	Pes cavus/scoliosis
Disability score^a^	5	6	5	6	4
MRI of the brain	NAD	NAD	NAD	NAD	NAD
Electrophysiological studies (NCS)	ND	ND	ND	ND	ND
Plasma amino acid levels	↑Arginine	↑Arginine	↑Arginine	↑Arginine	↑Arginine
Summary	Pyramidal weakness, cog/psych symptoms epilepsy, hearing impairment, sphincter disturbances, Pes cavus/scoliosis	Pyramidal weakness, cog/psych symptoms, epilepsy, sphincter disturbances	Pyramidal weakness, cog/psych symptoms, sphincter disturbances	Pyramidal weakness, Pes cavus/scoliosis	Pyramidal signs without weakness, cog/psych symptoms, Pes cavus/scoliosis

*UL, upper limbs; LL, lower limbs; PUL/PLL, proximal upper/lower limbs; DUL/DLL, distal upper/lower limbs; N, normal; NA, not applicable due to patient's condition; NAD, no abnormality detected; ND, not done; Cog./psych., cognitive/psychiatric*.

*Clinical signs severity: – Absent, + Mild, ++ Moderate, +++ Severe. Tendon reflexes: ↑ Increased tendon reflex. Extensor planter response: ↑ Unilateral, ↑↑ Bilateral, ↔ Mute, ↓ Flexor*.

a *Disability score: 0 = no functional handicap; 1 = no functional handicap, but signs at examinations; 2 = mild, able to run, walking unlimited; 3 = moderate, unable to run, limited walking without aid; 4 = severe, walking with one stick; 5 = walking with two sticks; 6 = unable to walk, requiring wheelchair; 7 = confined to bed*.

## Materials and Methods

### DNA Extraction

DNA was extracted from saliva collected from five patients and nine healthy parents and relatives using Oragene.Discover® DNA collection kits (DNA Genotek Inc.®, Ottawa, ON, Canada) according to the manufacturer's prepIT.L2P manual protocol.

### Whole-Exome Sequencing

Whole-exome sequencing (WES) was conducted for all five patients and one healthy parent. WES was performed on 150-bp paired-end reads using the Hiseq property of the NextSeq-500 sequencer (Illumina®, San Diego, CA, USA). Genomics Workbench (CLC Bio®, Aarhus, Denmark) was used for quality control and variant calling. Both probability-based and quality-based algorithms were used to identify single nucleotide variants (SNVs) and insertions/deletions (Indels). For gene/variant prioritization, a minimum depth of 30 × and 1% as the minor allele frequency cutoff were selected. Nonsense, frameshift, splice site, and predicted pathogenic missense variants were filtered according to the autosomal-recessive mode of inheritance predicted from the pedigree analysis.

### Sanger Sequencing

Sanger sequencing using the BIGDYE chemistry on an ABI3730 sequencer (Applied Biosystems®) was carried out on 13 affected and healthy family members to validate the WES results and confirm co-segregation between the pathogenic allele and the disease within the family. Chromas Lite® software (Technelysium®, South Brisbane, QLD, Australia) and Seqscape® (Applied Biosystems®) were utilized for sequence visualization and analysis.

## Results

**Figure 1:**
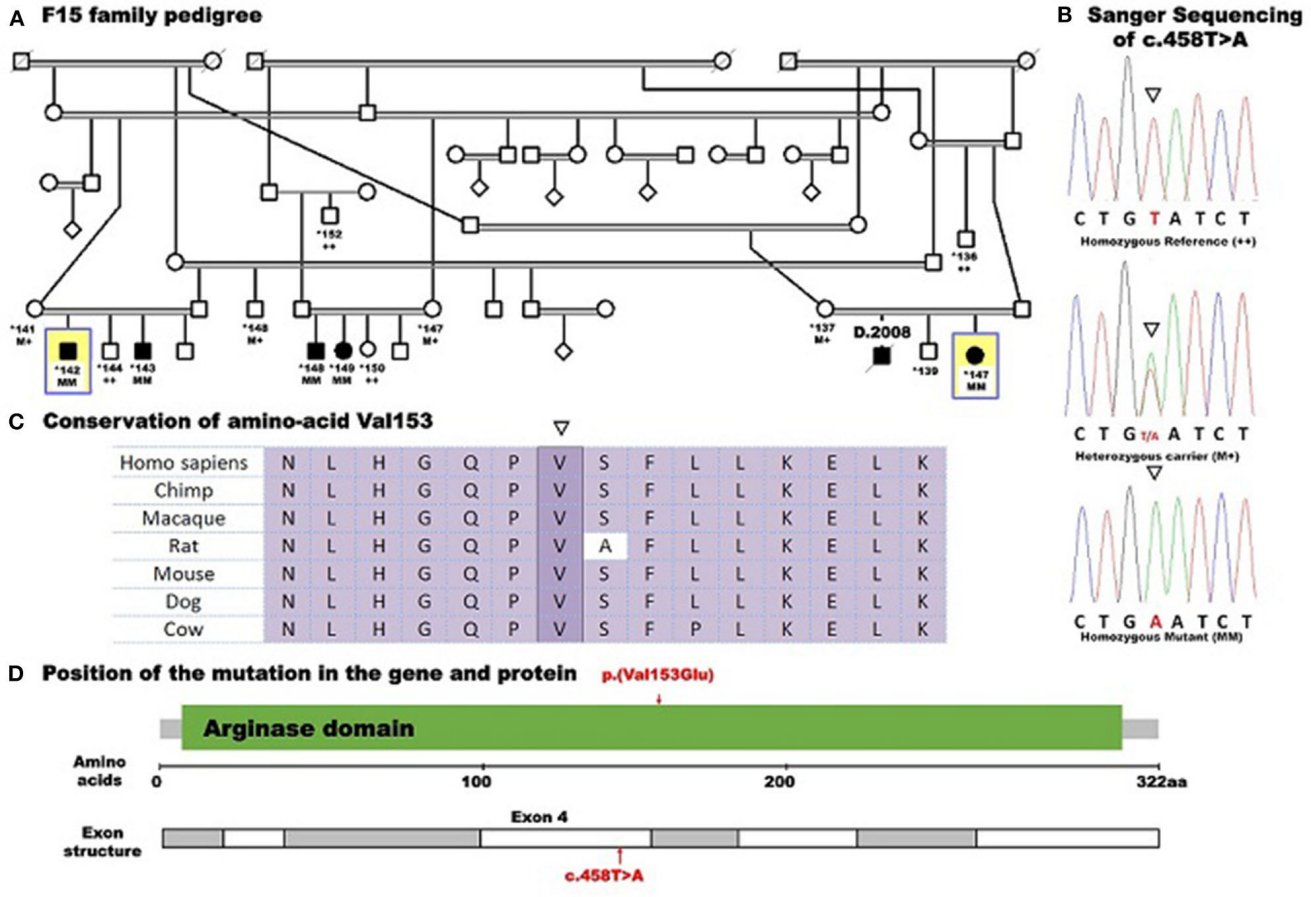
Pedigree of family F15 caused by a missense mutation in *ARG1* segregating with the disease distribution in the whole family presenting with spastic tetraplegia and mental retardation. Whole-exome sequencing was performed for five affected individuals (138, 142, 143, 148, and 149) and the mother (137) of patient 138. Example Sanger sequencing of the mutation in a patient (homozygous mutant), a heterozygous carrier, and a control homozygous reference allele with conserved amino acid sequence is shown. Pedigree symbols: *asterisk* indicates sampled individual; *yellow square* indicates the index patient. Genotype symbols: ++ Homozygous reference genotype; M+ Heterozygous genotype; MM Homozygous mutant genotype. Others are standard medical pedigree symbols.

After filtering out the common and non-coding variants, WES data analysis detected only one homozygous variant that was shared by all five patients, which was heterozygous in the healthy parent. This was a novel missense transversion variant [GRCh37 (NM_001244438.1): exon 4: g.131902487T>A, c.458T>A, p.(Val153Glu)] in the *ARG1* gene. The mutation was predicted pathogenic by five algorithms (SIFT = deleterious, Polyphen2 = probable damaging, mutation taster = disease causing, align GVGD = C35, and CADD score = 28.4) (Table 2). The variant was absent from gnomAD or dbSNP. Sanger sequencing revealed complete co-segregation between the pathogenic allele and the disease distribution, being a homozygous mutant in all the five patients and a homozygous reference or heterozygous carriers in nine related healthy individuals, including all parents who were available for sampling. The variant resulted in the replacement of the highly conserved (12 species) hydrophobic amino acid valine, located in the protein domain ureohydrolase, arginase subgroup, by the acidic amino acid glutamate (Figure 1). The moderately radical physiochemical difference [Grantham distance of 121 (0–215)] exists between the two amino acids. No other homozygous or compound heterozygous variants were found in genes known to be involved in other neurological disorders.

**Table 2:** Summary of the reported mutation in the *ARG1* gene.

**Gene**	**Exon/introninvolved**	**Predicted change**	**Protein change**	**Conseq-uence**	**Zygosity in patients**	**Conservation**		**Predicted pathoge-nicity**					**MAF**				**Novelty/reference**	**ACMGclassification**
						**PhastCons**	**PhyloP**	**SIFT**	**Polyphen2**	**Mutation Taster**	**Align GVGD**	**CADD Score**	**dbSNP**	**EVS**	**1000 Genomes**	**gnomAD**		
*ARG1*	Exon 4	NM_001244438 .1c.458T>A	p.(Val153Glu)	Missense	Homozygous	1.0	4.64	Deleterious	Probably damaging	Disease-causing	C35	28.4	Not found	Not found	Not found	Not found	Novel	Pathogenic: PS3, PM1, PM2, PP1-4

### Biochemical Assessment

Biochemical assessment of the amino acid levels in the serum of all the five patients showed an increased level of arginine. A second confirmatory test was performed for the proband only and showed an increased serum arginine level, confirming the first test.

### Counseling and Management

The family received genetic counseling, which included discussions with the senior family members (parents and grandparents) about consanguinity to provide the family a better understanding of the nature of their genetic disorders, increase their awareness about the role played by consanguinity in inheritance, and to eliminate any misperceptions. Good response to counseling regarding consanguinity was observed, particularly in the female members of the family, and a new generation is non-consanguineously married.

The patients were put on physiotherapy, with epilepsy treatment for the patients with seizures. However, most of the time, the family was faced with limited financial capabilities, which hindered their compliance with the follow-up and the physiotherapy.

A specialist was consulted for dietary modulation to start the patients on a restricted protein diet and arginine-free amino acid supplements. Two obstacles faced the use of special formulas with dietary restrictions: the availability of the formulas and the finance. The required special formulas were not available in Sudan, and attempts to obtain them from a neighboring country were not successful. In addition, the family was unable to afford either the cost of the formulas regularly from other countries or the necessary tests used for the monitoring of patients when started on treatment.

## Discussion and Conclusion

Arginase 1 deficiency can lead to a devastating neurological and cognitive dysfunction in children, though it is a potentially treatable condition (9). The age at onset was typical for all patients, and the clinical features of the five reported Sudanese patients, which included progressive spastic quadriplegia, regression of milestones, and epilepsy, are similar to what was reported about the presentation of a loss-of-function mutation in the arginase 1 enzyme (7, 10, 11). However, the five Sudanese patients maintained normal head circumference and normal physical growth despite the variable degrees of mental impairment witnessed in all of them. These features are different from what was reported from many Japanese and Chinese patients in whom microcephaly and poor physical growth were the major complicating features (1, 5, 12). The conservation of the nucleotide affected by the mutation reported in this study, (c.458T>A), as evidenced by two elevated conservation scores (PhastCons and PhyloP), and the prediction of pathogenicity by five algorithms (SIFT, Polyphen2, mutation taster and align GVGD, and CADD score) supported the significance of the variant which was classified as pathogenic according to the American College of Medical Genetics and Genomics (ACMG) classification (Table 2). The mutation (c.458T>A) is located in exon 4, and its pathogenic effect is in line with studies which have revealed missense mutations in certain conserved regions to be pathologic. This was further supported by a sequence analysis study which demonstrated clustering of the pathogenic missense variants in exons 1, 4, and 7. The involvement of these conserved regions in the catalytic activities of the enzyme makes mutations in these sequences likely to be pathogenic. This does not apply to truncating mutations that occur randomly along the gene, leading to non-sense-mediated protein decay and consequent hyperargininemia by complete loss of function (8, 13).

The high conservation of the mutated hydrophobic amino acid valine over mammalian species, its replacement by the acidic glutamate with radical Grantham distance existing between the amino acids, and the location of the mutated amino acid in the ureohydrolase domain, arginase subgroup further favor the pathogenicity of the change.

In the absence of other evidence of involvement of any other variants, the matching of the clinical phenotype and the biochemical profile of the patients to the published data, and the complete co-segregation of this variant with the disease, we considered the *ARG1* mutation as causative in this family.

In the Middle East region, there are some reported cases of hyperargininemia from Saudi Arabia, Palestine (14), and Bahrain (15). However, studies from Sub-Saharan Africa are extremely rare. This is the first study from Sudan to describe a novel missense mutation in the *ARG1* gene causing hyperargininemia, spastic quadriplegia, and cognitive dysfunction. Little is known about the incidence and the prevalence of the disease in Sudan, raising the need for more studies.

The Sudanese population has one of the highest overall consanguinity rates in the world with the highest first cousin/double first cousin marriages in the Arabic world (16, 17). Increasing evidence of augmenting AR inherited disorders including hyperargininemia is accumulating despite the scarcity of the statistics and the insufficiency of data about most of the genetic disorders in Sudan (18–22). This necessitates further studies to be conducted and campaigns to be launched in order to increase public awareness about the impact of consanguinity and its contribution to the shaping of the genetics of the individuals, families, and populations.

The lack of neonatal metabolic screening programs in Sudan leads to delayed diagnosis with the development of several neurological complications that include loss of sphincter control, inability to walk, and intellectual disability, which could have been avoided by early diagnosis and proper management (23, 24).

However, this study also highlighted deeper issues in the provision of the required care for patients with metabolic disorders in a limited-resource country like Sudan. Consequently, there is a necessity to conduct a larger-scale research project exploring some treatable metabolic disorders in Sudan in order to collect the required data that will be the basis for a national newborn screening program for these disorders. It draws attention to the importance of establishing specialized centers for metabolic disorders, which would increase the opportunity for early diagnosis and provide all the requirements for patients' care at an affordable cost. This will efficiently help decrease the disability as a consequence of potentially treatable causes in Sudanese children, especially with the advent of more advanced options of therapy like rescue mRNA therapy in addition to the existing treatment options (25, 26).

## Data Availability Statement

The datasets presented in this study can be found in online repositories. The names of the repository/repositories and accession number(s) can be found at: https://www.ncbi.nlm.nih.gov/, VCV000916528.1.

## Ethics Statement

The studies involving human participants were reviewed and approved by The Ethical Committee of Medical Campus, University of Khartoum, Sudan according to the recommendations of the Helsinki Declaration. Written informed consent to participate in this study was provided by the participants' legal guardian/next of kin. Written informed consent was obtained from the individual(s) and/or minor(s)' legal guardian/next of kin for participation in the study and for the publication of any potentially identifiable images or data included in this article.

## Author Contributions

LE, GS, AEA, AB, ME, and MI formulated and designed the study and contributed to the interpretation of data. AB, GS, AEA, ME, and MI granted funds and participated in manuscript editing, review, and critique. LE, IM, AH, ME, MS, AY, RS, MK, and ASIA contributed to the clinical, radiologic, and biochemical data collection and interpretation. LE, GS, MK, and AY contributed to the bioinformatic analysis. All authors read the manuscript, assented to be submitted, and accepted and agreed to be responsible for all aspects of this work.

## Conflict of Interest

The authors declare that the research was conducted in the absence of any commercial or financial relationships that could be construed as a potential conflict of interest.
